# Circulating Regulatory B-Lymphocytes in Patients with Acute Myocardial Infarction: A Pilot Study

**DOI:** 10.3390/jcdd10010002

**Published:** 2022-12-22

**Authors:** Igor Volodarsky, Sara Shimoni, Dan Haberman, Vita Mirkin, Yakov Fabrikant, Tal Yoskovich Mashriki, Adi Zalik, Jacob George

**Affiliations:** 1 The Heart Institute, Kaplan Medical Center, Rehovot 7661041, Israel; 2 Hadassah Medical School, the Hebrew University, Jerusalem 9112102, Israel; 3 The Hematology Institute, Kaplan Medical Center, Rehovot 7661041, Israel

**Keywords:** myocardial infarction, acute coronary syndrome, regulatory B cells, inflammation

## Abstract

Background: Inflammation plays on important role in plaque instability and acute coronary syndromes. The anti-inflammatory effects of B-regulatory lymphocytes (B-regs) in atherosclerosis was tested mainly in animal models with inconclusive results. Herein, we studied for the first time, levels of circulating B-regs in patients with acute myocardial infarction (MI). Methods: We examined circulating levels of B-regs by flow cytometry in 29 patients with recent ST-segment elevation MI and 18 patients with stable angina pectoris (SAP) and coronary artery disease. We re-assessed B-reg levels on average 4 months later. Results: The mean level of CD20+ cells was similar in patients with MI and patients with SAP (*p* = 0.60). The levels of CD24^hi^CD38^hi^ cells among CD20+ cells were 5.7 ± 4% and 11.6 ± 6% in patients with MI and SAP, respectively, (*p* < 0.001). The level of CD24^hi^CD38^hi^ B-regs remained related to acute MI after correcting for age, gender, and risk factors. Circulating levels of CD24^hi^CD38^hi^ B-regs in patients with MI did not change significantly at follow-up in a small patient groups (*p* = 0.408). Conclusions: Circulating B-regs are reduced in patients with MI compared to patients with SAP. This finding may shed further light on the inflammatory pathophysiologic factors related to plaque rupture.

## 1. Introduction

Atherosclerosis is a multifactorial disease with multiple genetic and environmental risk factors. During the past decades, the crucial role of inflammation in the development and complications of atherosclerosis has been established. The inflammatory process affects the intima media of the coronary arteries and contributes to the development of atherosclerosis, coronary artery disease (CAD), and late complications such as plaque rupture and acute coronary syndrome (ACS) [1,2]. Chronic inflammatory response is mediated by both the innate and the adaptive immune system at all stages of atherogenesis [3,4].

In the early stages of atherosclerosis, the retention of LDL in the arterial intima triggers the inflammatory response. Several domains are involved in the process. These domains act as altered-self antigens attracting macrophages, lymphocytes, and other immune cells [5]. Plaque inflammation is further amplified and sustained by recruitment and activation of the adaptive immune system, leading to plaque rupture.

B cells have recently come to the forefront of the research on atherosclerosis, with conflicting results. Several studies showed that global B-cell depletion therapy attenuates both the development and progression of atherosclerosis in mouse models [6]. Other studies showed that B-cell depletion in mice is atheroprotective [7]. This suggests that B cells are not a uniform population of cells. B cells are responsible for adaptive immunity; they secrete immunoglobulins that bind to unprocessed antigens. B2 lymphocytes are the most abundant B cells in spleen and lymphoid organs. They produce IgG, which is antigen-specific and acts in a Th-dependent way. Depleting B2 cells, in gene-modified atherosclerosis-prone mice resulted in attenuation of atherosclerosis. Adoptive transfer of B2 cells aggravated atherosclerosis [8]. B1a B cells produce predominantly IgM-type antibody [9,10]. IgM-type antibodies bind to oxidized LDL and are present in apoptotic cells or within atherosclerotic plaques [11,12]. IgM-type antibodies targeting modified LDL particles attenuated atherosclerosis in several studies in mice [13,14,15].

“B-regulatory lymphocytes” are subtypes of B lymphocytes with a role in immunosuppressive activity [16,17,18]. The immunosuppressive activity of B cells was first noticed in delayed hypersensitivity reactions [19] and later in experimental autoimmune encephalitis (EAE), where their absence was shown to induce EAE and a subset of B cells could ameliorate EAE and experimental autoimmune arthritis [20,21]. During the last decade, several subpopulations of these cells were studied in mice and in humans [22,23]. The best described subsets in humans are CD24+CD38+ cells (transitional cells or immature cells) and CD24+CD27+ cells (B10 cells, corresponding to murine CD5^+^CD1d^hi^ cells) [24,25]. Human CD24^hi^CD38^hi^ B cells were demonstrated to suppress T-helper-1 cell proliferation, production of pro-inflammatory cytokines by them, conversion of naïve T cells to Th1 and Th17, and even induce conversion of other T-cell lineages into T-regs lymphocytes [26].

Previously the anti-atherogenic role of IL-10 and TGF-β was attributed exclusively to T-regs. However, B-regs produce TGF- β and IL-10, too [27,28,29,30]. Recent studies addressing the effect of IL-10-producing B cells on atherosclerosis in mice showed equivocal results [31,32].

There is a paucity of data on the importance of circulating B-regs in human atherosclerosis. Recently, Liu et al. described low levels of CD19^+^CD24^hi^CD38^hi^ B cells in patients with severe coronary atherosclerosis referred to coronary bypass surgery [33]. They found a negative correlation between levels of CD19^+^CD24^hi^CD38^hi^ and severity of atherosclerosis. We aimed to assess the levels of B-regs (CD24^hi^CD38^hi^ and CD24+CD27+) in patients with acute ST elevation myocardial infarction (STEMI) and to compare them to patients with stable angina pectoris (SAP) and to controls with no evidence of CAD. To our knowledge, this is the first study to report on the circulating levels of B-reg subpopulations in patients with STEMI.

## 2. Materials and Methods

The study group included 29 patients with STEMI hospitalized in the intensive cardiac care unit and 18 patients with stable angina with evidence of coronary artery disease, which underwent elective coronary angiography in the Heart Center of Kaplan Medical Center. Nine patients with MI and nine patients with stable disease had history of previous MI (11.4 ± 5.9 years and 10.6 ± 6.8 years prior to recruitment into the study, respectively). Two patients with MI and five patients with stable disease had history of coronary artery bypass graft surgery. The patients in SAP had no acute coronary event at least 2 years prior to enrollment. Patients with acute or chronic inflammatory or infectious conditions and patients with hematopoietic or oncologic diseases were excluded. Patients who were hemodynamically unstable and patients with glomerular filtration rates lower than 30 mL/min were also excluded. This study was approved by the Kaplan Medical Center institutional ethics committee (KMC-01710-14) and all patients provided written consent forms.

### 2.1. Quantification of Regulatory B Cells by Flow Cytometry

Circulating regulatory B cells were defined as CD20+CD24^hi^CD38^hi^ and CD20+CD24+CD27+ cells and quantified by flow cytometry. Twenty ml of peripheral blood was drawn from each patient in the MI group and stable angina group; the blood was drawn 3 (range 0–7) days after catheterization. The blood sample was diluted with Dulbecco’s Phosphate Buffered Saline (PBS) without calcium and magnesium (Biological Industries, Cromwell, CT, USA; 02-023-1A) 1× (ratio 1:1). The diluted samples were subjected to density gradient separation (ratio 2:1) (Axis-shield; Lymphoprep™ 1114547) and centrifuged. After centrifugation, the PBMC layer was collected and washed with PBS.

1 × 10^6^ cells were incubated at 4 °C in the dark for 30 min with:

1. Mouse anti-human CD24 (eBioscience; 17-0247-41), + mouse anti human CD38 (eBioscience; 12-0388-41) + mouse anti-human CD20 (eBioscience; 11-0209-41).
2. Mouse anti-human CD24 + mouse anti human CD27 (eBioscience; 12-0279-41) + mouse anti-human CD20.

Isotype controls were used. After incubation, cells were washed with PBS and analysed by FACS (BD FACSCalibur™). Levels of CD24^hi^CD38^hi^ cells and CD24^int^CD38^int^ were assessed.

### 2.2. Intracellular IL10 Staining

For intracellular IL-10 staining, cells were fixed with 2% pFA- for 10min at 4 °C, and permeabilized with 0.2% Tween20- for 15min at 37 °C. After incubation cell were washed with PBS.

1 × 10^6^ cells were incubated at 4 °C in the dark for 30 min with: Rat anti-human IL-10 (eBioscience; 53-7108-41). Isotype controls were used. After incubation, cells were washed with PBS and analysed by FACS (BD FACSCalibur™).

### 2.3. Serum IL-10 Assessment

We studied the levels of IL-10 in sera of the patients enrolled in the study. The levels were examined using ELISA test (RayBiotech; ELH-IL10).

### 2.4. Data Analysis

Continuous data is presented as mean ± SD. Categorical data are presented as absolute numbers with their respective percentages. Chi-square or Fisher’s exact test was used for categorical variables and Student’s *t* test was used for continuous variables. When the data were not normally distributed, Mann–Whitney U test was used.

Pearson was used to assess correlation between CD24^hi^CD38^hi^ cells and age, troponin, and IL-10 levels. Chi-square or Fisher’s exact test were used to assess correlation between CD24^hi^ CD38^hi^ cells and gender, presence of hypertension, diabetes mellitus, and hyperlipidemia. Binary logistic regression analysis was performed in order to adjust for the differences between the groups in regard to baseline characteristics (STEMI vs. SAP). Paired *t* test was used to compare B-reg levels at baseline and follow-up. All the above analyses were considered significant at *p* ≤ 0.05.

## 3. Results

The population included 29 patients with recent ST-segment elevation MI (mean age 61 ± 10 years, 90% male) and 18 patients with stable angina (mean age 64.8 ± 6, years, 94.4% male). All patients with STEMI underwent revascularization within 90 min. All patients with SAP had coronary artery disease, and 16 of them underwent revascularization. In two patients with SAP, no revascularization was performed due to chronic total occlusion. In the MI group and SAP group, the levels of B-regs were measured 3 (range 0–7) days after coronary angiography. Patient baseline characteristics are presented in Table 1. There was no difference in baseline characteristics between patients with STEMI and patients with SAP. The number of vessels with >50% stenosis was similar in patients with STEMI and patients with stable angina.

**Figure 1:**
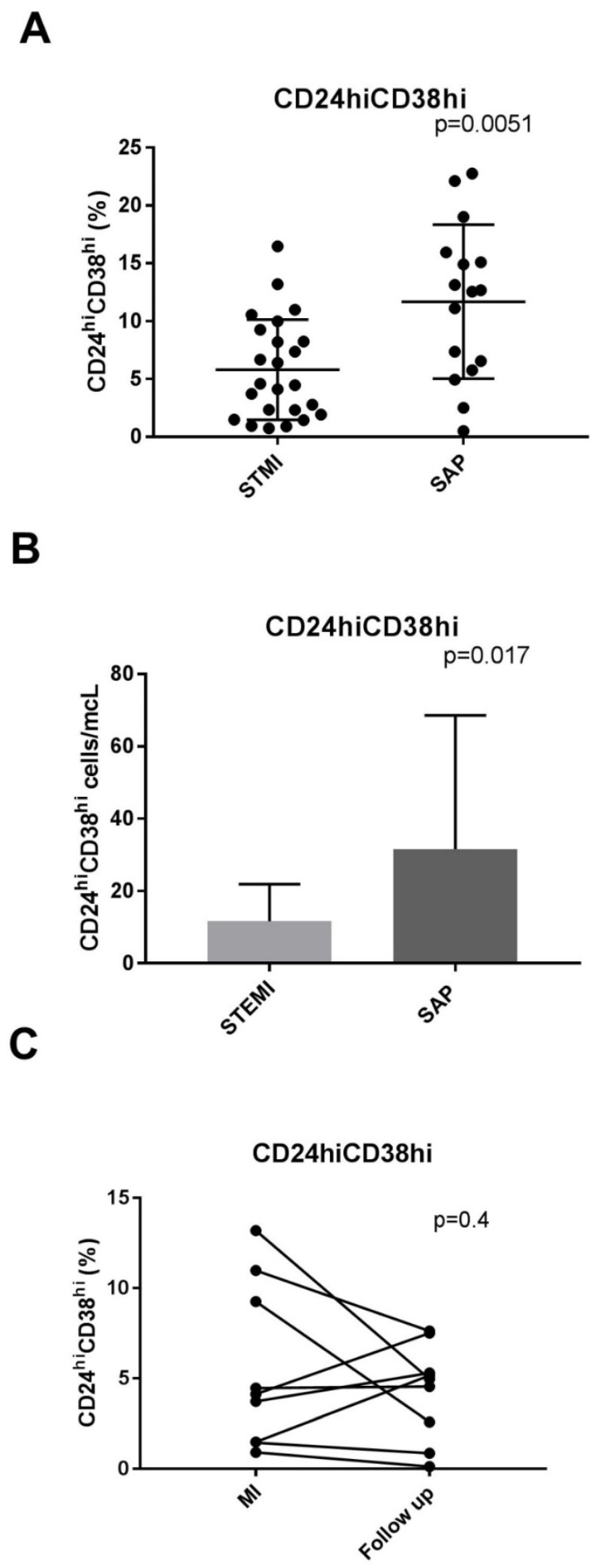
CD20+CD24^hi^CD38^hi^ B cells from STEMI patients are lower compared to SA patients. (**A**) CD24^hi^CD38^hi^ as a percent of CD20+ cells in STEMI patients and stable angina patients. Scatter plots show mean percentages of B-reg cells in the peripheral blood of STEMI and SA patients. (**B**) CD24^hi^CD38^hi^ cells/mcL (absolute number) in STEMI patients and stable angina patients. (**C**) CD24^hi^CD38^hi^ cells in patients with STEMI, acute phase compared to follow-up.

**Figure 2:**
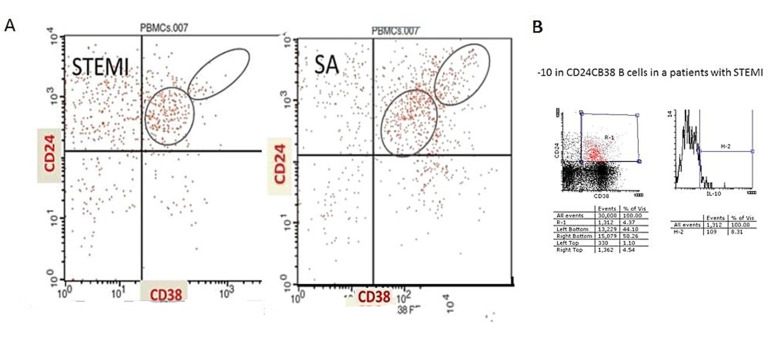
(**A**) Circulating CD24+CD38+ in a patient with stable angina and a patient with acute myocardial infarction. PBMCs isolated from patients with STEMI and SAP were stained directly ex vivo for the expression of CD20, CD24, and CD38. Representative flow cytometry plot of B cell subset gating showing CD20+CD24^hi^CD38^hi^ and CD20+CD24^int^CD38^int^. (**B**) Intracellular IL-10 expression in CD24+CD38+ cells. Representative flow cytometry plot of IL-10 in CD24+CD38+ cells in a patient with STEMI.

The percentage of CD20+ cells in patients with myocardial infarction was 6.96 ± 2.6% and 6.79 ± 3.3% in patients with stable angina *p* = 0.6, Table 2. The level of CD24^hi^CD38^hi^ cells among the CD20+ cells was 11.6 ± 6% in patients with stable angina, while it was as low as 5.7 ± 4% in patients with myocardial infarction (*p* = 0.0051, Figure 1a). The absolute levels of CD20+CD24^hi^CD38^hi^ cells were 8 (2–16) cells/mcL in patients with ST elevation MI and 18 (7–44) cells/mcL in patients with stable angina (*p* = 0.017, Figure 1b). There was no difference in the levels of CD20+CD24^int^CD38^int^ between the groups (34 ± 13% and 3720%± in MI patients and stable angina patients, respectively, *p* = 0.68). A representative flow cytometry evaluation of CD20+CD24^hi^CD38^hi^ and CD20+CD24^int^CD38^int^ cells in a patient with acute MI and a patient with SA is shown in Figure 2a. No significant difference was found between patients with myocardial infarction and SAP in the level of CD24+CD27+ cells (20 ± 10% and 23 ± 6%, in MI patients and stable angina patients, respectively, *p* = 0.4).

On binary logistic regression, after correcting for age, gender, presence of hypertension, diabetes mellitus, hyperlipidemia, and smoking history, only the level of CD24^hi^CD38 ^hi^ B-regs was related to the presence of acute MI (OR 0.881, 95% CI 0.819–0.949, *p* = 0.001).

No correlation was seen between CD24^hi^CD38^hi^ and age, gender, presence of hypertension, diabetes mellitus, or hyperlipidemia (*p* = 0.788, *p* = 0.143, *p* = 0.715, *p* = 0.624, and *p* = 0.98, respectively). No correlation was found between the level of CD24^hi^CD38^hi^ cells and infarct size as defined by maximal level of troponin during the hospitalization (r = −0.04, *p* = 0.86).

Intracellular staining of IL-10 was performed in 5 patients with MI. We found that in patients with MI, 10% of cd24+cd38+ cells were positive for IL-10. Figure 2b shows a representative flow cytometry evaluation of intracellular IL-10 in 2 patients with MI.

Serum IL-10 levels were assessed in all patients with STEMI. The level of IL-10 in the MI group was 30.67 ± 12.66 pg/Ml. No correlation was seen between CD24^hi^CD38^hi^ cells and IL-10 (*p* = 0.55)

We randomly selected nine patients from the MI cohort and examined the levels of the subgroups of B-regulatory lymphocytes on average 4 months (range 2–6 months) after MI. As can be seen in Table 3 (Figure 1c), there was no significant change in the level of CD24^hi^CD38^hi^ cells (*p* = 0.408). As can be seen in the figure, the levels of CD24^hi^CD38^hi^ cells did not change or were even lower compared to baseline. There was a small decline in the level of CD24+CD27+ cells. However, the significance of this finding is not clear since in these nine patients the level of CD24+CD27+ cells was relatively high in the acute MI phase.

## 4. Discussion

In this study, we demonstrated for the first time that the levels of CD24^hi^CD38^hi^ B-reg cells were significantly lower in patients with acute MI compared to patients with stable CAD. In the small group of patients studied, the levels of circulating CD24^hi^CD38^hi^ cells did not change during follow-up. There was no difference in the level of naive B cells (CD24^int^CD38^int)^. No differences in the levels of CD20+ B cells and CD24+CD27+ B-reg cells were observed between the groups.

In humans, B-regs were studied mainly in autoimmune diseases. Two main B-reg subsets were studied: (1) the B10 regulatory B cells (CD24hiCD27) that produce IL-10 and suppress effector CD4+T cells, monocytes, and dendritic cells. These cells are increased in autoimmune diseases such as rheumatoid arthritis and systemic lupus erythematosus and decreased in graft versus host disease, newly diagnosed graves diseases, and pemphigus. (2) The transitional or premature B cells are the CD24^hi^CD38^hi^ cells. In healthy individuals, CD19+ CD24^hi^CD38^hi^ B cells limit the differentiation of naïve CD4+ T cells into Th1 and Th17 populations and convert effector CD4+ T cells into CD4+FoxP3 + T-regs with suppressive capacity. Low levels of circulating CD24+CD38+cells were seen in patients with active autoimmune diseases such as active RA. In addition, this subset may be functionally compromised in patients with RA and SLE. These diseases are also associated with increased risk of cardiovascular disease and accelerated atherosclerosis; however, the exact mechanism for this risk is not known.

The role of B-cells in atherosclerosis was studied in various animal models and is controversial [7,8]. In these models, spleen- and lymph-node-derived B-regs were assessed and comprise mainly of the B10 B-reg subtype. Gjurich et al. showed in an L-selectin-deficient Apoe^−/−^mice model of atherosclerosis a decreased in aortic B1a and B-reg population, suggesting a correlation between atherosclerosis and decreased B1A and B-reg levels [34]. Recently two studies showed conflicting results regarding the atheroprotective function of B-regs in murine models. Sage et al. demonstrated that male LDLR^−/−^ mice irradiated and reconstituted with 80% B-cell deficient bone marrow and 20% bone marrow from IL-10-deficient mice had no difference in the size and cellular contents of the lesion compared with 20% wild-type bone marrow despite marked reductions of IL-10 in B-regs^31^ On the other hand, a study by Strom et al. found that the CD21^hi^CD23^hi^CD24^hi^ B-reg subset was increased in the draining lymph nodes of ApoE^−/−^ mice. Adoptive transfer of these cells into female ApoE^−/−^ mice attenuated neointima formation in response to perivascular-collar-induced carotid artery injury. Inhibition of IL-10 using a neutralizing Ab or adoptive transfer of B cells from IL-10-deficient mice prevented lymph-node-derived B-cell atheroprotection [32]. The reason for the differences in the results of these studies may be related to the type of animal model, different mechanisms of atherosclerotic development, or different sources of regulatory B lymphocytes. It has been suggested that the action of B-regs is dependent on the inflammatory environment [35].

There is very limited data on the role of regulatory B cells in atherosclerosis in humans. We found a decreased level of circulating CD24^hi^CD38^hi^ cells in patients with AMI and no change in B10 B-regs. The percentage and the absolute number of CD24^hi^CD38^hi^ cells was significantly lower in patients with ACS compared to patients with stable coronary disease, suggesting the possible role of B-regs in acute stage of plaque rupture. Recently, Liu et al. assessed CD19+CD24hiCD38hi levels in patients with severe CAD before coronary bypass surgery [33]. They report a significantly lower CD19+CD24^hi^CD38^hi^ level in patients with CAD. They also show a statistically significant correlation with Gensini score; however, the correlation coefficient is low (r = 0.283). There is no information on the clinical status of the patients and a comparison between stable and unstable coronary syndrome. We did not perform Gensini scores in our study; however, we did not find a correlation between the number of vessels with significant stenosis and B reg levels. There is a significant difference in the inflammatory response in stable patients with atherosclerosis and patients with acute MI due to ruptured, unstable plaque with inflammation of the fibrous cap that leads to thrombus formation [36]. Inflammation plays a key role in promoting plaque vulnerability via thinning the fibrous cap, enhancing the influx of lipids and expanding the lipid core, and stimulating neoangiogenesis. Various inflammatory markers, including CRP and nonspecific markers of inflammation, are related to plaque instability [37,38]. Van Dijk et al. assessed the cellular components of the adaptive immune response in a biobank of human aortas and found unique vulnerable lesions consisting of B cells and occasional plasma cells in tertiary follicle-like structures [39]. They did not assess regulatory B cells; however, this and other findings suggest that B cells play a role in plaque rupture.

Both T-regs and B-regs contribute to maintaining immune tolerance. B-regs facilitate activating T-regs but then disappear, while T-regs continue to be operational [40]. Recently it was shown that T-reg cells are twice as frequent in coronary thrombi compared with peripheral blood [41]. These cells were characterized by clonal restriction in peripheral blood and coronary thrombi from patients with ACS. Patients with ACS were shown to exhibit an altered T-cell repertoire evident by an increase in IFN-γ levels produced by CD4+CD8– cell populations [42]. These findings are supported by the observation that Th1 subsets are expanded in patients with ACS [43]. In addition, our group has shown that compared with patients who have stable angina, patients with ACS who have a similar extent of coronary atherosclerosis have a reduced number of naturally occurring Tregs [44]. Regulatory T cells were shown to be atheroprotective in several reports by suppressing atherosclerosis-associated inflammation [45,46]. Moreover, the suppressive properties of isolated T-regs, namely their ability to attenuate proliferation of effector Th cells, were compromised in patients with ACS [44]. Since CD24^hi^CD38^hi^ cells are shown to limit the differentiation of naïve CD4+ T cells into Th1 and Th17 populations and convert effector CD4+ T cells into CD4+FoxP3+ T-regs with suppressive capacity, it is possible, that lower levels of CD24^hi^CD38^hi^ cells are related to a less efficient conversion of CD4 T cells into T-reg cells, thus enhancing plaque inflammation with potential consequent rupture. Macrophages and mast cells infiltrates are seen in regions of plaque rupture [47,48,49]. There is a cross-talk between macrophages and mast cells to B-reg cells in various tissues [50,51]. B-regs inhibit macrophage activation, negatively regulate the antigen presenting cell function of macrophages, and decrease the production of pro-inflammatory cytokines such as IFN-g and TNF by macrophages [52]. B-cell-activating factor (BAFF), produced by macrophages, induces B-reg cells and there is a synergy between AngII and BAFF in inducing IL-10 production by B cells; however, excess BAFF inhibits B-regs [53,54]. Mast cells can support or depress B-reg development and B-regs can alter mast cell function in various tissues [55,56]. Mast-cell-produced IL-5 is important in maintaining the population of IL-10+ B-reg cells in peripheral lymphoid tissues to depress contact hypersensitivity. However, the interaction between CD24^hi^CD38^hi^ B-regs and macrophages and mast cells in unstable plaque is not known.

Regulatory B cells dampen the allergic response. In humans, among the main chronic allergic diseases such as asthma, allergic rhinitis, food allergies, atopic dermatitis, and insect venom allergies, the frequencies of different B-reg subsets varies compared with healthy individuals. A reduced frequency of CD24^hi^CD38^hi^ B-regs was found in atopic dermatitis patients, and the disease severity was inversely correlated with B-regs [57]. There are several reports of increased frequency of hypertension and coronary disease in patients with allergy [58]. In addition, some recent studies have suggested that, at least in a subset of patients, cellular mediators of allergic inflammatory responses, in particular, eosinophils, basophils, and mast cells, may also play a pathogenetic role in coronary plaque instability [59]. Whether B-regs are involved in this process needs to be investigated.

We did not find a difference in the B10 (CD24+CD27+) B -regs between patients with MI, stable angina, and the control group. Although there was a small increase in these cells in patients during follow-up, the significance of this finding is not clear. We did not observe a difference in the levels of IL-10 between patients with acute MI and the control group. There is controversial data on circulating IL-10 levels in patients with unstable coronary disease. The levels are related to timing of sampling, degree and extent of heart injury, and co-morbidities [60,61,62]. It is known that the B10 cell function is mainly related to IL-10 production. In addition, and most importantly, IL-10 can be secreted by other cells. Although most studies focused on IL-10-mediated regulation by B-regs, and have been extensively reviewed, other mechanisms, including direct contact with T cells, GITR-mediated regulatory T-cell maintenance, IL-35 and TGF-β production, and adenosine generation via CD73, have also been well documented [63]. The close correlation between IL-10 and B-regs is less significant in humans as compared to mice [24].

The inflammatory response after myocardial infarction includes a number of different players such as neutrophils, monocytes, macrophages, dendritic cells, and lymphocytes as well as cardiomyocytes, endothelial cells, fibroblasts, and the interstitium. Myocardial reperfusion following coronary intervention also exacerbates this pro-inflammatory response [64], so we cannot define whether the change in CD24^hi^CD38^hi^ is a primary phenomenon or secondary to ischemia/reperfusion injury. Although the majority of patients with SAP underwent revascularization, the type and magnitude of the inflammatory response is different. The stable number of CD24^hi^CD38^hi^ cells in a small number of patients at follow-up suggests that these changes are not only due to acute MI and revascularization; however, this needs to be validated. Activated B cells promote cytokine secretion and may participate in the pathological process of myocardial fibrosis after AMI [65]. Experimental depletion of B cells by blocking CD20 can inhibit pressure-overload-induced cardiac remodeling and dysfunction in mice [66]. However, preliminary reports showed that B-regs are important in post MI remodeling. Jiao et al. showed in a murine MI model that B-regs limit ventricular remodeling after MI through decreasing CCR2-mediated monocyte recruitment and mobilization [67]. Unfortunately, we cannot comment on the correlation of B-reg levels and LV remodeling in our patients.

Our study has several limitations. The first is the relatively small number of patients in each study group. However, this is the first study that assessed circulating B-regs in patients with STEMI and, in addition, in regard to CD24^hi^CD38^hi^ cells, the difference is statistically solid. The follow-up study included only nine patients. However, as mentioned in the results section, there was no change or even reduction in CD24^hi^CD38^hi^ cell levels in the majority of patients that were low in the acute phase. We included only patients with STEMI and did not include the NSTEMI group in order to keep the study group a homogenous patients group. This was designed as a hypothesis-generating study and as such we wanted to look for a positive signal and accordingly have made fine-tuned calibration of the FACS analysis markers. The trends evident in the study clearly point to such a signal that would be tested comprehensively in subgroups. Please note that we have already published a paper on regulatory T cells in patients with MI [46].

A study of the functionality of B-regs has not been performed in this study since it is a pilot study in patients with MI and the assays are not well documented and reproducible as with T-regs. We did perform an intracellular IL10 staining.

In conclusion, this is the first study demonstrating the reduction in circulating subsets of B-regulatory cells in patients with acute coronary syndrome as compared to stable patients. This exploratory study may shed additional light on the complex factors governing plaque destabilization and subsequent rupture.

## Figures and Tables

**Table 1:** Patient characteristics.

	STEMI *n* = 29	SAP *n* = 18	*p* (STEMI vs. SAP)
Age	61 ± 10	65 ± 7	0.3
Male gender	26	17	0.9
Hypertension	15	13	0.28
Diabetes mellitus	11	6	0.9
Hyperlipidemia	21	17	0.124
Troponin (ng/mL)	23.79 ± 24.27		
Number of vessels >50% stenosis (1/2/3 vessel disease (%)	28/26/46	17/50/33	0.128
History of MI *n* (%)	9(31)	9 (50)	0.32
History of revascularization	2(7)	5(27)	0.09

STEMI = ST elevation myocardial infarction, SAP = stable angina pectoris.

**Table 2:** Circulating regulatory B lymphocytes in patients with acute myocardial infarction and patients with stable angina pectoris.

	STEMI *n* = 29	SAP *n* = 18	*p* (STEMI vs. SAP)
CD20+(%)	6.96 ± 2.6	6.79 ± 3.3	0.6
CD24^hi^CD38^hi^(%)	5.7 ± 4%	11.6 ± 6%	0.0051
CD24^hi^CD38^hi^ \(cells/mcL)	8 (2–16)	18 (7–44)	0.017
CD24^int^CD38^int^(%)	34 ± 13	37 ± 20	0.68
CD24+CD27+(%)	20.8 ± 10.7	23.7 ± 6.1 ^a^	0.4
CD24+CD27+ (cells/mcL)	33 (18–53)	43 (27–100)	0.3
IL-10	30.67 ± 12.7		

^a^, *n* = 10; STEMI = ST elevation myocardial infarction; SAP = stable angina pectoris.

**Table 3:** Comparison between levels of B-reg subgroups in patients after MI in acute and convalescent phase (*n* = 9).

	Acute Phase MI	Convalescent Phase	*p*-Value
CD20+(%)	7.98 ± 2.9	8.14 ± 2.86	0.465
CD24^high^Cd38^high^ (%)	5.7 ± 4	4.3 ± 0.9	0.408
CD24^int^Cd38^int^ (%)	26 ± 14	32 ± 12	0.23
CD24+CD27+ (%)	25.8 ± 9.34	21.7 ± 10.18	0.0493

MI = myocardial infarction, B-reg = regulatory B cells.

## Data Availability

Data are available upon request.

## Abbreviations

MI	myocardial infarction
SAP	stable angina pectoris
B-regs	B-regulatory lymphocytes
CAD	coronary artery disease
WBC	white blood cells
LVEF	left ventricular ejection fraction
